# Long-term adaptation of the influenza A virus by escaping cytotoxic T-cell recognition

**DOI:** 10.1038/srep33334

**Published:** 2016-09-15

**Authors:** Rutger G. Woolthuis, Christiaan H. van Dorp, Can Keşmir, Rob J. de Boer, Michiel van Boven

**Affiliations:** 1 Theoretical Biology, Utrecht University, Utrecht, The Netherlands; 2 National Institute for Public Health and the Environment, Bilthoven, The Netherlands

## Abstract

The evolutionary adaptation of the influenza A virus (IAV) to human antibodies is well characterised. Much less is known about the long-term evolution of cytotoxic T lymphocyte (CTL) epitopes, which are important antigens for clearance of infection. We construct an antigenic map of IAVs of all human subtypes using a compendium of 142 confirmed CTL epitopes, and show that IAV evolved gradually in the period 1932–2015, with infrequent antigenic jumps in the H3N2 subtype. Intriguingly, the number of CTL epitopes per virus decreases with more than one epitope per three years in the H3N2 subtype (from 84 epitopes per virus in 1968 to 64 in 2015), mostly attributed to the loss of HLA-B epitopes. We confirm these observations with epitope predictions. Our findings indicate that selection pressures imposed by CTL immunity shape the long-term evolution of IAV.

IAV persists by continuously escaping pre-existing immunity in the population. Most attention has been on the evolution of surface proteins hemagglutinin (HA) and neuraminidase (NA) that form the main targets of neutralising antibodies^1^,^2^,^3^. Antibody mediated immunity is subtype specific and lasts for 2–7 years due to rapid evolution of the antigenic sites on the HA and NA proteins^4^,^5^,^6^. IAV also elicits CTL immune responses^7^,^8^, which reduce viral spread within the host by killing infected cells. As with memory B cells, memory CTLs mount a fast immune response upon recognition of epitopes years after the primary infection^9^,^10^, such that individuals with pre-existing CTLs develop less severe disease^11^,^12^. CTLs also provide heterosubtypic immunity^13^,^14^, which could be an attractive feature for universal vaccines^15^.

Viruses escape CTL recognition by mutating amino acid residues within CTL epitopes. Such immune escape mutations play an important role in the within-host dynamics of chronic pathogens (e.g. HIV) and are also observed during acute IAV infection^16^,^17^. While immune escape mutations in IAV cripple the virus^18^,^19^, these mutations can persist in a prolonged infection^20^, and at the population level despite the high polymorphism of human leukocyte antigen (HLA)^21^,^22^. Recently, positive selection in CTL epitopes has been shown in the nucleoprotein (NP) by comparing human and swine viruses in a phylogenetic analysis^23^. Many CTL epitopes have been identified in IAV^24^,^25^, but a framework capturing the dynamics of CTL epitopes in all proteins over long evolutionary time is lacking. Here we analyse historical and contemporary IAV sequence data spanning the period 1932–2015, using 142 empirically confirmed CTL epitopes known to date^26^,^27^ (Supplementary Tables S1, S2 and S3; Methods).

## Results

### Antigenic cartography based on CTL epitopes

**Figure 1:**
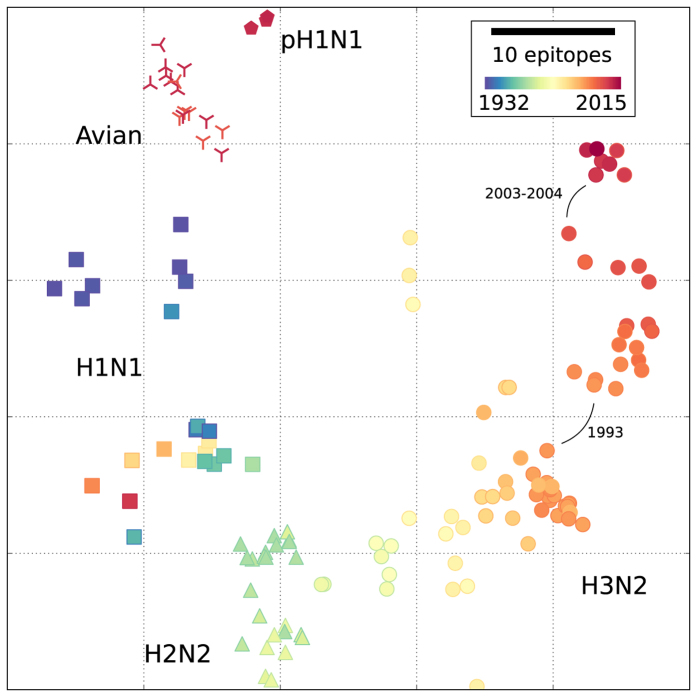
CTL epitope evolution in the influenza A virus. Antigenic map of 295 representative influenza A viruses spanning the period 1932–2015 (□H1N1, △H2N2, ○H3N2, 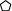 pH1N1) based on 134 CTL epitopes. The H3N2 subtype has evolved extensively over the period 1968–2015, while the H2N2 and H3N2 viruses circulating in the late 1960s are antigenically close. Recent avian viruses are superimposed independently onto the antigenic map (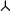 H5N1, 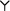 H7N9 and 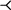 H9N2), using the 134 CTL epitopes of human IAV origin. The map is constructed using multi-dimensional scaling (MDS) based on Jaccard distances, explaining 93% of the antigenic distances (Methods). Scale bar denotes expected differences in the number of epitopes; colours indicate collection year of the virus.

We combine 295 representative human IAVs and the compendium of CTL epitopes into an antigenic map that tracks the long-term evolution of CTL epitopes in IAV across the H1N1, H2N2 and H3N2 subtypes (Fig. 1). Each virus contains a subset of the CTL epitopes (Supplementary Fig. S1 and Supplementary Fig. S2), with on average 74 epitopes per virus (summed over all class I HLAs). In total, we find 134 out of the 142 epitopes in these viruses, of which 24 are conserved in the study period (marked in Supplementary Table S2). At seven loci (positions in the proteome) we find more than one confirmed epitope, i.e. at these loci epitope variants have mutated at some point in time to another epitope variant.

As a measure of immune similarity we use the Jaccard index, defined as the number of epitopes shared by a pair of viruses divided by their number of unique epitopes (Methods). This measure is attractive biologically as it is based on overlaps of epitopes between viruses, and counts any mutation in an epitope as a CTL escape. Multidimensional scaling (MDS) based on Jaccard distances yields a map in which the distance between any pair of viruses represents the expected number of different epitopes (Fig. 1). The map accurately visualises the expected cross-immunity between viruses, even across subtypes (R^2^ = 0.93, Supplementary Fig. S3). We find similar results using Manhattan distances (Supplementary Fig. S3). Similar maps based on antibody-mediated immunity can only be constructed for each subtype separately, because hemagglutination inhibition assays are subtype specific^4^,^5^.

In the CTL antigenic map, a time directional evolutionary path runs from the early A/1932 (H1N1) to the recent A/2015 (H3N2), with the H2N2 subtype located between the H1N1 and H3N2 subtypes (Fig. 1). The highly reassorted 2009 pandemic H1N1 (pH1N1) virus is distinct from all other viruses, and is genetically (Supplementary Fig. S4) and antigenically (Fig. 1) most closely related to the early twentieth century H1N1 viruses.

The early H3N2 viruses (1968–1970) are remarkably close to the previously circulating H2N2 viruses (1957–1967; Fig. 1). Conversely, the H3N2 subtype has evolved by more than 35 CTL epitopes over the past 46 years (Fig. 1), with noticeable antigenic jumps in 1993 and 2003–2004. The near-continuous H2N2-H3N2 transition results from the fact that only HA and polymerase basic 1 protein (PB1) were reassorted, while HA carries few known CTL epitopes and PB1 is a highly conserved protein. The antigenic jump in 1993 results from a R384G mutation in NP, affecting four epitopes that are restricted by the HLA alleles B*44, B*08:01, B*27:02, and B*27:05^28^. In 2003–2004, five epitopes mutated in the NP, M1, and PA proteins. The CTL antigenic map differs completely from a map based on amino acid sequences of the viruses (Supplementary Fig. S4). Here subtypes are clearly separated, mostly due to the variable HA and NA proteins. Excluding HA and NA from the amino acid map decreases the difference with the epitope map, in particular with respect to the subtype clustering (Supplementary Fig. S4). Nevertheless, there are still important differences between the antigenic and genetic maps, like the absence of the antigenic jumps in the amino acid map. Overall, IAV gradually drifts away from ancestral viruses by escaping CTL epitopes.

Not all epitopes are expected to impose equal immune pressures on the IAV. First, epitopes vary in immunodominance due to heterogeneity in HLA-allele frequencies in the population. However, when weighting the epitopes with the corresponding HLA supertype frequencies of five main ethnicities, we find that the distances between viruses are hardly affected (Supplementary Fig. S5). Similarly, some epitopes are intrinsically immunodominant by evoking stronger immune responses than others, e.g., due to differences in precursor frequencies^29^. Since the relative immunodominance of epitopes is not known, weighting for their exact contribution is impossible. However, when we assign random weights to the relative contribution of the different epitopes, we find that even with substantial immunodominance the results are unaffected (Supplementary Fig. S6). Thus, while individual immune-responses might be affected by dominant CTL epitope escapes, the analyses show that at the population level the pattern of viral CTL epitope evolution in Fig. 1 is robust.

The antigenic cartography allows us to investigate avian IAVs that cause human infections and may pose a pandemic threat^14^,^30^,^31^. To characterise the antigenic relatedness of avian viruses to human viruses in the context of human CTL immunity, we superimpose representative avian viruses of the subtypes H5N1, H7N9 and H9N2 onto the antigenic map (Fig. 1; Methods; Supplementary Table S4). The avian viruses are more similar to pH1N1 viruses than to recent H3N2 viruses, suggesting that prior infection with a pH1N1-like virus is likely to yield more CTL immunity to the avian viruses than infection with recent H3N2 viruses. This illustrates that positioning of a virus in the antigenic map is possible on the basis of its sequence, enabling assessment of the extent of pre-existing CTL immunity in the human population.

### Escape of CTL mediated immunity

We hypothesise that IAV evolution is in part driven by adaptation to pre-existing CTL immunity. To test this hypothesis, we compile a data set of 62 HLA binding peptides of IAV origin that do not elicit a CTL response (Methods; Supplementary Table S3). With the exception of a small number of early H1N1 viruses in the period 1932–1935, the confirmed epitopes are significantly more variable than the non-immunogenic HLA-binding peptides (Supplementary Fig. S7 and Supplementary Fig. S8). Additionally, the viruses are located considerably closer together in a map obtained using distances based on the non-immunogenic peptides than in the antigenic map (Supplementary Fig. S9). The difference in the evolutionary rates between CTL epitopes and non-immunogenic peptides suggests that there is selection for escape of CTL epitopes.

**Figure 2:**
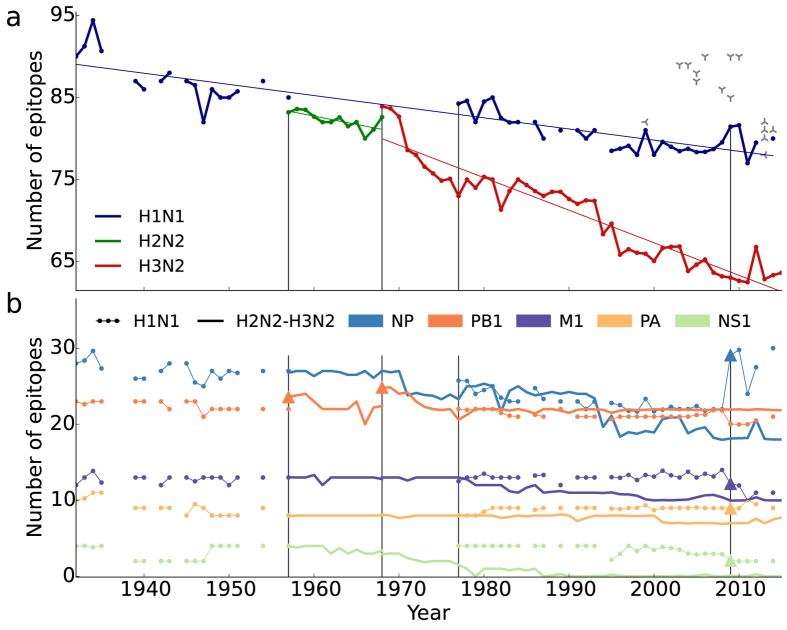
Decrease in the number of CTL epitopes over time. (**a**) The number of CTL epitopes per virus decreases with 0.14 ± 0.01, 0.20 ± 0.06, and 0.40 ± 0.02, epitopes per year (SEM) in H1N1, H2N2, and H3N2, respectively (Methods). The number of CTL epitopes in avian viruses is larger than in recent human viruses (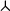 H5N1, 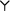 H7N9 and 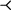 H9N2). (**b**) Protein specific CTL epitope dynamics showing a decrease in the number of epitopes in NP, matrix 1 protein (M1) and non-structural protein (NS1). The other IAV proteins are laid down in Supplementary Fig. S10. Reassortment events resulting in the appearance of novel proteins are marked by triangles. Subtype replacements are marked by vertical lines. The analysis is based on 7,347 whole-proteome sequenced viruses (Methods).

To substantiate this further, we use 7,347 whole-proteome sequenced viruses (Methods), and find that the total number of epitopes per virus decreases over time (Fig. 2a). In H3N2 approximately one epitope is lost every two-and-a-half years, and in H1N1 approximately one epitope is lost every eight years. Since these time series are ancestrally dependent, we also consider the annual (relative) change in the number of epitopes (Methods), and reject neutral evolution for H3N2 (P = 0.043, Wilcoxon signed-rank test). We cannot distinguish neutral from directed evolution for H1N1 and H2N2, perhaps because the decrease is too small for H1N1 and the time series is too short for H2N2. The number of epitopes decreases most markedly in NP, while remaining almost constant in PB1 (Fig. 2b and Supplementary Fig. S10).

The decrease in the number of CTL epitopes could possibly be due to an underrepresentation of recently emerged epitopes which may be less likely to be identified than CTL epitopes in old viruses. However, the number of non-immunogenic peptides is constant over time (Supplementary Fig. S11), suggesting that the decrease is unlikely the result of observational bias.

**Figure 3:**
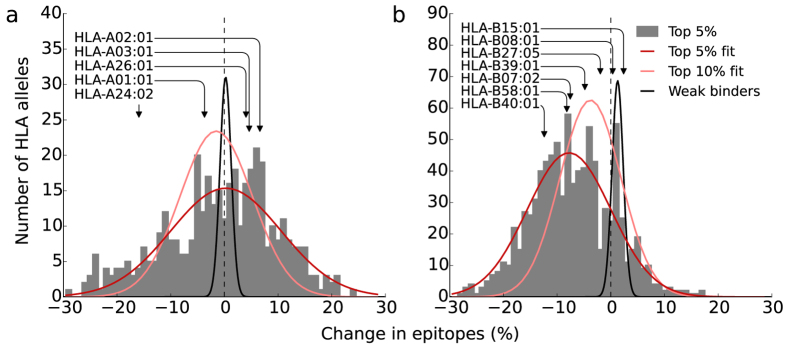
Decrease in the number of predicted epitopes. The change in number of top 5% strongest binding peptides in NP of H3N2 viruses for HLA-A (**a**) and HLA-B (**b**) alleles is shown over the period 1968–2015 (histograms; Methods). These peptides decrease in number for the large majority of HLA-B alleles (P = 0.006; mean change 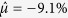 (95% CI: −15.6%, −2.6%) over the study period, Methods), but not for HLA-A alleles (P = 0.46). Despite the high level of heterogeneity in binding motifs, we find a decrease in the number of top-binders for a broad range of HLA-B alleles (Supplementary Fig. S12). The position of the twelve HLA supertype representatives are indicated with arrows. Fitted Gaussian curves indicate the decrease calculated for the thresholds ≤5%, ≤10% (top binders), and ≥50% (weak binders).

In addition, we obtain an independent test by computationally predicting potential CTL epitopes. These predictions are free from any observational bias that may be present in the set of empirical epitopes. In the analysis we use prediction tools based on HLA-peptide binding affinities^32^, using a suite of binding thresholds and focussing on the top 5% binders. With this threshold 88% of the empirical epitopes is present in the predicted set (Supplementary Fig. S12 and Supplementary Fig. S14). For NP in H3N2, which harbours most of the empirical epitopes (Fig. 2b), we find a decrease in the number of strongly binding peptides for the large majority of HLA-B alleles (Fig. 3). We find a similar though non-significant trend when combining the NP, M1, and PB1 proteins in H3N2 (Supplementary Fig. S14). These observations are in agreement with a significant decrease in the number of empirical epitopes restricted to HLA-B alleles (Supplementary Fig. S15). The decrease is two-fold larger in HLA-B restricted epitopes compared to HLA-A restricted epitopes. The predictions show that the decrease in the number of empirical epitopes per virus (Fig. 2a) is not restricted to the empirical set of epitopes.

**Figure 4:**
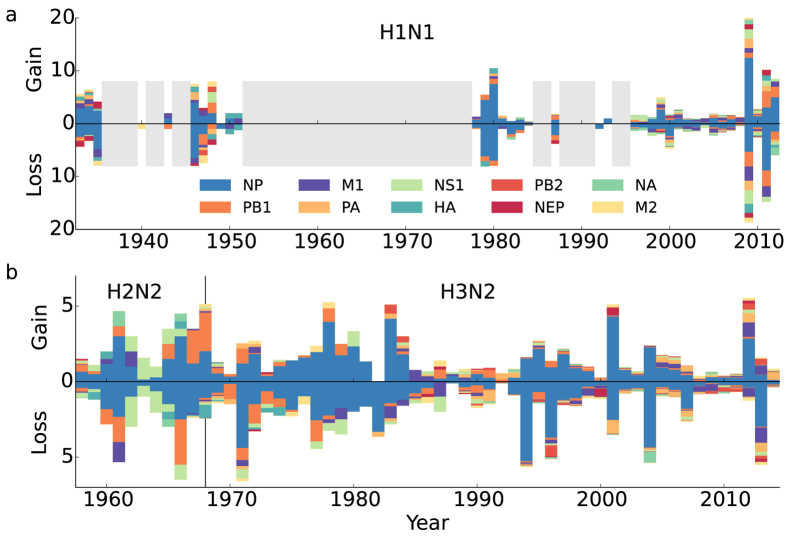
CTL epitope dynamics. Epitopes appear and disappear continuously in H1N1 (**a**) and H2N2-H3N2 (**b**), with the highest turnover in NP. Within years epitope gains and losses are correlated, due to annual variation in genetic drift. We calculate for each epitope the difference in the fraction of viruses harbouring the epitope in a given year and the year before. Summing over all positive (negative) differences of the fractions gives the total gain (loss).

The loss of CTL epitopes is the long-term net result of a continuous and rapid turnover of epitopes (Fig. 4). In the short-term, genetic drift of the virus results in similar (and correlated) losses and gains of epitopes (Fig. 4). Here we calculated the losses and gains of the epitopes weighted by their frequency in the 7,347 viruses. Of the two proteins containing most epitopes, NP has a higher turnover of epitopes than the more conserved PB1, in H1N1 as well as in H3N2. As in the antigenic map we find no unusual losses or gains during the H2N2-H3N2 transition. The turnover of epitopes shows that only a fraction of the escapes results in a lower number of epitopes per virus.

## Discussion

By combining CTL epitope data with IAV sequence data, we provide evidence that IAV evolves to escape preexisting cellular immunity in the population. Namely, mutations accumulate more rapidly in epitopes than in non-immunogenic peptides, and the total number of epitopes decreases over time (Supplementary Fig. S7 and Fig. 2).

Mutations in epitope sites can result in two different types of escape. First, T-cell receptor (TCR) escapes prevent memory T cells to recognise the mutated epitope. In this case the virus circumvents preexisting memory responses against the epitope in the population, but can trigger the expansion of other immune responses upon infection. The second type are escapes that prevent loading of the epitope. This happens when the mutation either prevents HLA binding or the processing of the epitope. In this case the host cannot elicit any T-cell response against the epitope. Combinations of these escapes are also possible when the mutation lowers the TCR as well as the HLA binding affinity.

In our analysis we treat both escapes equally, since the exact impact of the mutations is unknown. This might be an oversimplification, because some of the mutations may hardly affect the binding with either TCR or HLA. However, experiments have shown that CTL escape mutations usually affect the immune response^16^,^17^,^18^,^28^. In addition, the decrease over time in the number of strongly binding predicted peptides indicates that the overall HLA binding affinity decreases as well, confirming that mutations in epitopes lower the HLA binding. Therefore, we believe that most mutations do affect the CTL response and argue that the simplification of treating any mutation in epitopes as escape is justified.

The adaptation to preexisting CTL immunity that we observe is in agreement with a recent study showing positive selection at CTL epitope sites in NP by a comparison of evolutionary rates in swine and human IAVs^23^. Using a phylogenetic analysis the authors find an increased rate of epitope mutations on the trunk of the tree in human compared to swine IAVs. In line with this, we find that the change in epitopes is larger than the change in non-immunogenic peptides (Supplementary Fig. S7). In addition, we find that CTL epitope loci disappear over time, which is a direct indication of adaptive evolution (Figs 2 and 3). In agreement with Machkovech *et al*.^23^, the decrease in the number of CTL epitopes is most pronounced in NP, although we see similar trends in other proteins.

At this stage the impact of the lower number of CTL epitopes in IAV is unknown, as there are no studies done showing changes in the virulence of influenza. Correlating incidence data with epitope escapes is complex, since other factors, like vaccination and the rapid antigenic drift due to neutralising antibodies^5^, interfere with the CTL mediated drift. While additional experiments have to unravel the phenotypic effect of the adaptation, circumventing preexisting CTL immunity could in principle affect the severity of disease, since the lack of preexisting cellular immunity results in more severe infection^11^,^12^,^33^.

We speculate that the decrease in the number of CTL epitopes has been ongoing since the global selective sweep of the internal proteins of IAV in the late 1800s^34^. Possibly, the number of CTL epitopes could return to a high value when zoonotic rearrangements introduce proteins that have not undergone evolutionary adaptation to human cellular immunity^23^. Examples are the increase of the number of epitopes in PB1 of H3N2 viruses in 1968, and the increase of the number of epitopes found in NP in the pandemic H1N1 viruses in 2009 (Fig. 2b). Underpinning this further, we find a high number of epitopes in the avian viruses, especially in H5N1 (Fig. 2a).

Escaping humoral immunity releases IAV from antibody-mediated selection pressures generated by viruses circulating in the recent past^4^,^5^. This is not the case for cellular immune responses that have the potential to impact the long-term evolution of the virus. Since human IAVs seem to be adapted to CTL immune responses, novel viruses with unadapted reassorted internal genes would be at a selective disadvantage in the human population. This may explain why emerging IAVs with reassorted internal genes historically have only been successful when introduced in viruses that have also reassorted their HA and/or NA genes.

## Methods

### Complete virus set

We collected human IAV sequences (H1N1, H2N2, and H3N2) from the GISAID EpiFlu database from the period 1932–2015 (www.gisaid.org; July 2015)^26^. We selected viruses for which all protein sequences (except PB1-F2) are available and of which the length of each protein was not shorter than 20 amino acids of the consensus protein length. Moreover, we excluded any virus with sequences containing ambiguous amino acids (X, J, B). We excluded all H1N1 viruses collected in the year 1976, because these viruses originated from an exceptional swine flu outbreak in a military basis in New Jersey^35^. Moreover, A/Victoria/36/1988 was excluded, because this virus is a rare reassorted virus, and A/Canada/720/2005 (H2N2), because the subtype H2N2 did not circulate in 2005. This procedure resulted in 3,050 H1N1 (including pH1N1), 65 H2N2, and 4,213 H3N2 viruses.

### Characteristic virus set

For computational reasons we compiled a subset with characteristic viruses spanning the period 1932–2015, and used this set for constructing the antigenic map. For the H1N1 and H3N2 subtypes, we included all viruses used by Westgeest *et al*.^2^ and Bedford *et al*.^5^, together with A/Michigan/14/2014 (H3N2), A/Pennsylvania/44/2014 (H3N2), A/Sweden/1/2015 (H3N2) and A/Michigan/02/2015 (H3N2) to cover recent years, and included H2N2 viruses. In total, the characteristic set contains 295 viruses (Supplementary Table S1), of which 48 are H1N1, 52 are H2N2, 185 are H3N2, and 10 are pH1N1.

### Epitope compendium

We downloaded the epitope data from IEDB by using the filters ‘human’ as host organism and ‘Influenza A virus’ as source organism and selecting ‘Linear epitopes’, ‘MHC Class I’ and ‘T Cell Assays’ (www.iedb.org; September 2015)^27^. Next, we gather unique epitopes with all corresponding immune assays. Some epitopes are embedded in longer epitopes belonging to the same HLA supertype^36^. In these cases, we kept only the embedded (shorter) epitopes. Thereafter, we removed epitopes with length larger than 12 amino acids, because the exact (shorter) epitope within these longer peptides are not reported. We also excluded epitopes that were not present in any of the IAVs in the complete set described above. Finally, we characterised the candidate epitopes either as immunological epitopes or as non-immunogenic peptides. The candidate epitopes with at least one reported non-negative T-cell assay are added to our epitope compendium, while candidates having negative T-cell responses in all assays are classified as non-immunogenic peptides. The list of 142 epitopes and 62 non-immunogenic peptides is provided in Supplementary Tables S2 and S3 respectively.

### Jaccard distance

As a measure for the antigenic distance between two viruses we use a scaled adjusted Jaccard distance, reflecting the expected number of different epitopes between viruses *i* and *j*,


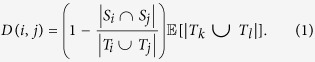


Here *S*_*i*_ and *S*_*j*_ represent the epitopes in viruses *i* and *j*, with 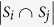 the number of epitopes that two viruses *i* and *j* have in common. *T*_*i*_ and *T*_*j*_ represent the loci of viruses *i* and *j* carrying a functional epitope, with 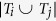 the number of unique loci in viruses *i* and *j*. Furthermore, 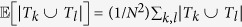 is the expected number of unique epitope loci, with *N* the total number of viruses. To account for the fact that two different alleles at the same locus is a unit difference in epitopes, we deviate from the classical Jaccard distance by dividing by |*T*_*i*_ ∪ *T*_*j*_| instead of |*S*_*i*_ ∪ *S*_*j*_|. Notice that |*S*_*i*_| = |*T*_*i*_|, because a virus can at most have one epitope at a given locus, and that the difference between the classical Jaccard distance, e.g., as used elsewhere^37^, and our distance is small, since only a few epitopes in our compendium contain multiple alleles.

### Manhattan distance

As an alternative to the Jaccard distance, we computed the Manhattan distance by counting the fraction of all possible epitope loci differing between pairs of viruses. In contrast to the Jaccard distance, this measure also counts epitope loci that are absent in both viruses, and therefore depends on the size of the epitope compendium. Additionally, the Jaccard index better counts the number of epitopes that would be recognised when encountering a second virus after infection with the first virus of a pair. Therefore, we believe that the Manhattan distance is less appropriate immunologically. Nevertheless, we found that there is an almost one to one relation between the two distance measures (Supplementary Fig. S3), such that the results do not change qualitatively, and to a large extent quantitatively, when using the Manhattan distance.

### Pairwise proteome distance

We concatenated the protein sequences for each virus and perform a global alignment using the Clustal Omega package^38^. The whole-proteome pair-wise distances of the aligned viruses are calculated with Clustal Omega, which uses the *k*-tuple distance metric (with values between 0 to 1).

### Antigenic map construction using multi-dimensional scaling

We follow Smith *et al*.^4^ by using geometric multi-dimensional scaling (MDS) to represent *N* viruses as elements of a low dimensional Euclidean space (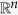), while preserving the distances between viruses as much as possible. Positions 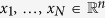 for the *N* viruses are calculated by minimising the error function *E*.


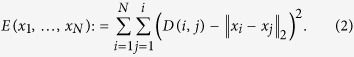


Here 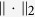 is the Euclidean norm on the low dimensional space 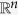. We minimised the error for different initial configurations to ensure that the global minimum is found. With increasing target dimension *n*, the error decreases strongly and the variance in the distances captured (R^2^) increases (Supplementary Fig. S3).

### Superimposing avian viruses

From GISAID, we collected 21 avian viruses of the H5N1, H7N9, and H9N2 subtypes. To assess the extent of human cellular immunity against avian viruses, we determine their position in the antigenic map given the positions of the human viruses, i.e. we minimise *E* in Eq. 2 for each of the 21 avian viruses independently. See Supplementary Table S4 for an overview of CTL epitopes in the three avian subtypes causing human infections.

### Predictions

In the HLA-peptide binding prediction analyses, we use all unique HLA alleles available in the netMHCpan 2.8 prediction tool^32^, and measure the relative change in the number of top-binders over a period of time. We focus on the top 5% and the top 10% strongest binders, and use the >50% (weakest binders) as comparative control (% is relative to a large set of peptides used in netMHCpan). Most empirical epitopes belong to the 5% top binders (Supplementary Fig. S12).

### Statistics

The slopes of the decrease in number of epitopes are calculated by linear regression (Fig. 2a). Since the number of epitopes depends on the number of epitopes in previous years, we also calculated the difference in the number of epitopes from year-to-year (excluding missing years) and used the conservative Wilcoxon signed-rank test (one tailed) to estimate the significance of the decrease. The analysis with non-immunogenic peptides was performed similarly (Supplementary Fig. S11).

Since we expected that the change in the number of binders (**x**) to be alike for HLA alleles with similar binding motifs, we used a statistical model that corrects for these dependencies when determining the significance of the mean decrease per HLA allele. We assume that 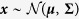, where 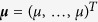, and **∑** is the covariance matrix describing the above mentioned dependencies. We calculate **∑** using the similarities between the binding motifs as follows: a HLA allele *i* can be represented as a binary string *b*^*i*^ indexed by all 9-mers *p* so that 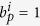 if *p* is predicted to bind *i*, and 0 otherwise. We then choose 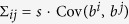 for some constant *s* > 0, and obtain maximum-likelihood estimates for the mean decrease *μ* and scaling parameter *s*. This model is selected in favour of a model that assumes independence of the HLA alleles (*P* < 10^−12^, likelihood-ratio test). The maximum likelihood estimates of the mean decrease and corresponding 95% confidence intervals are given in Fig. 3.

## Additional Information

**How to cite this article**: Woolthuis, R. G. *et al*. Long-term adaptation of the influenza A virus by escaping cytotoxic T-cell recognition. *Sci. Rep.* **6**, 33334; doi: 10.1038/srep33334 (2016).

## Supplementary Material

Supplementary Information

Supplementary Dataset 1

Supplementary Dataset 2

Supplementary Dataset 3

Supplementary Dataset 4
