# Supplementary material for: Long-term adaptation of the influenza A virus by escaping cytotoxic T-cell recognition
Source: Sci Rep. 2016 Sep 15;6:33334. doi: 10.1038/srep33334 (PMC5024124; doi:10.1038/srep33334)
Supplement: Supplementary Information [file srep33334-s1.pdf]

Supplementary information

Long-term adaptation of the influenza A virus by escaping cytotoxic T-cell recognition

Rutger G. Woolthuis, Christiaan H. van Dorp, Can Keşmir, Rob J. de Boer, &

Michiel van Boven

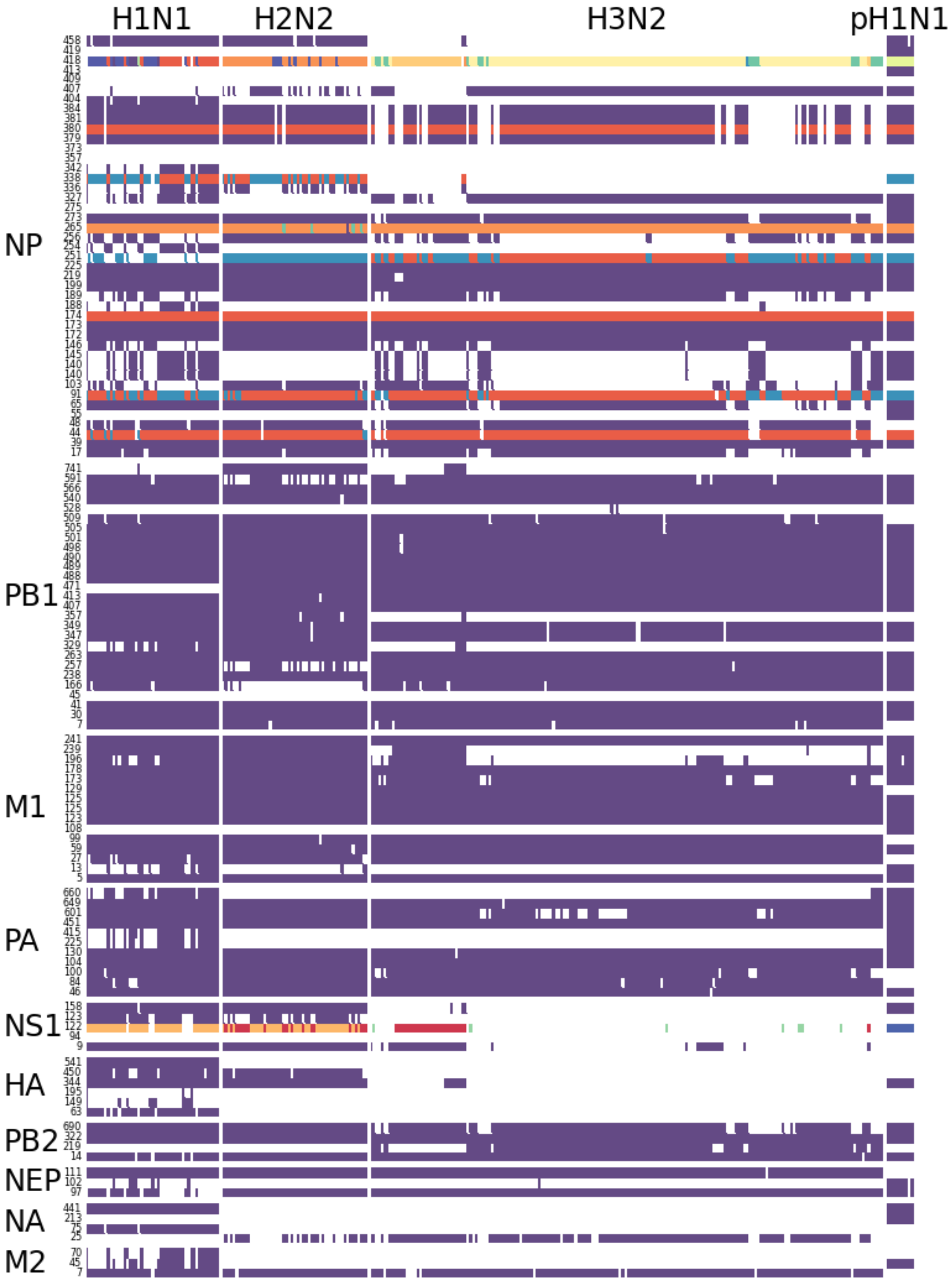

Supplementary Figure S1   Overview of CTL epitopes by subtype.

See next page for caption.

Supplementary Figure S1 Overview of CTL epitopes by subtype.

Coloured rows indicate the presence of epitopes (vertically ordered by protein and protein position) in the 295 viruses of the characteristic set (horizontally ordered by subtype and collection year), using different (arbitrarily chosen) colours if multiple epitope variants are present at a locus (white: no epitope present). Single mutations can result in multiple epitope escapes, as observed in the NP_{379}, NP_{380}, NP_{381} and NP_{384} epitopes (subscript indicates the starting position of the epitope). Some epitopes re-emerge after a period of absence, e.g. the NP_{91} and NP_{251} epitopes. NP_{418} is the most variable epitope.

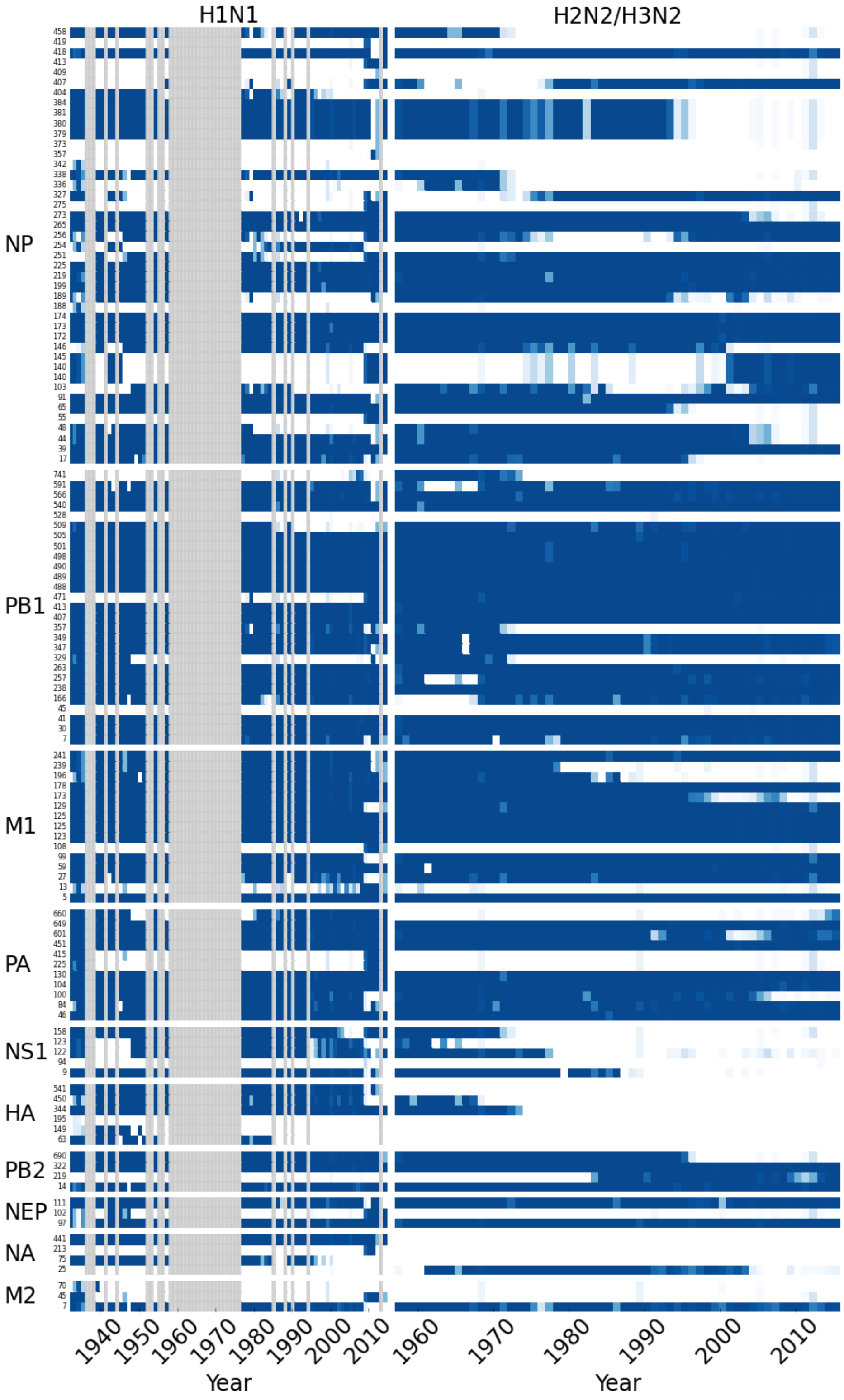

Supplementary Figure S2 Overview of CTL epitopes by year.

See next page for caption.

Supplementary Figure S2 Overview of CTL epitopes by year.

The fraction of viruses harbouring the CTL epitope (ordered vertically) in given year (ordered horizontally; white: epitope not present; grey: no data available). H1N1 and H3N2 are plotted separately, because these subtypes have co-circulated since 1977. We use all whole-proteome sequenced viruses available in the GISAID EpiFlu database (3,050, 65, and 4,213 viruses for H1N1, H2N2, and H3N2 respectively).

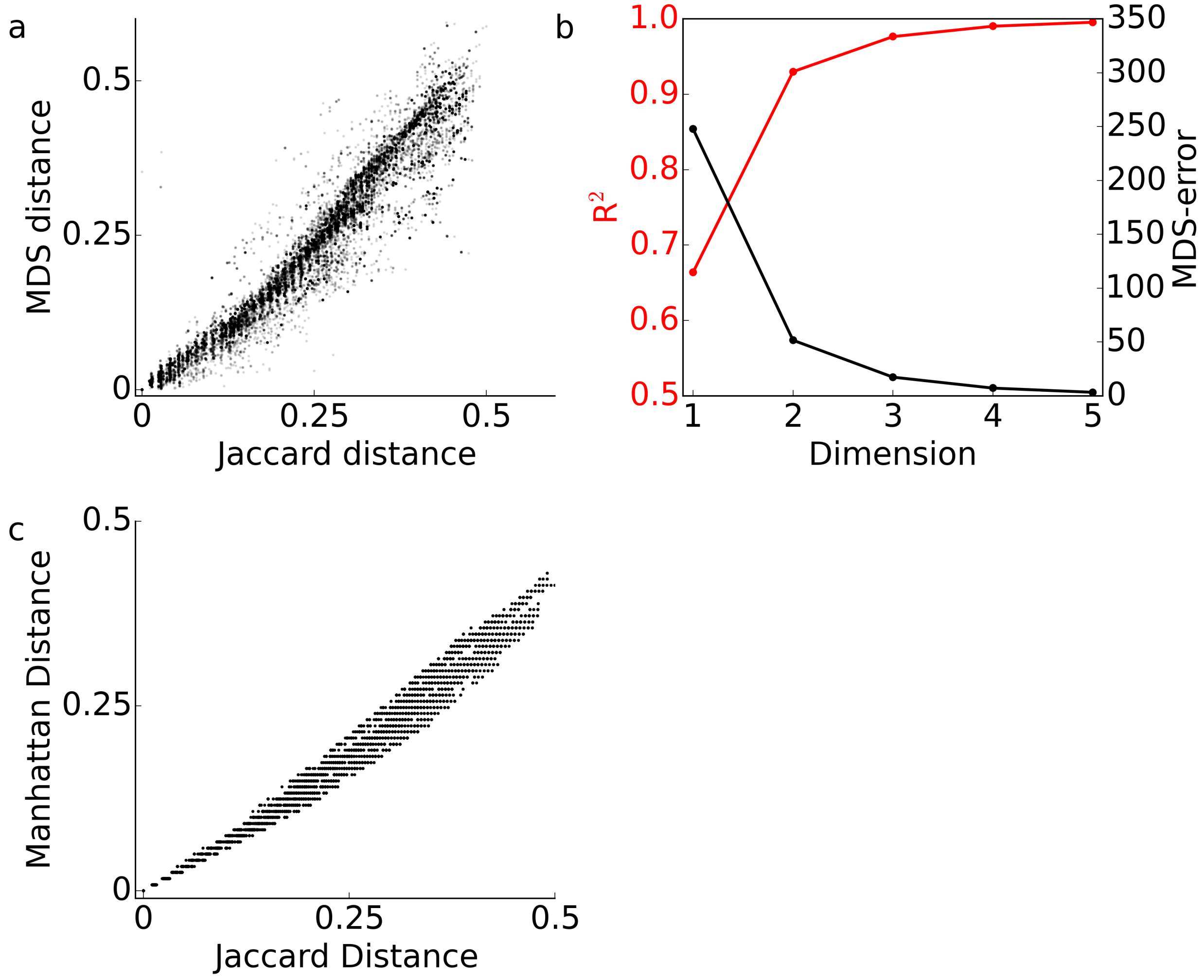

Supplementary Figure S3 Variance explained by the antigenic map and comparison with Manhattan distance.

(a) The antigenic map of Fig. 1 accurately visualises antigenic distances, since the two-dimensional projected MDS pairwise distances capture most of the variance in the multidimensional Jaccard distances ($R^2 > 0.93$). (b) R^2 saturates quickly with increasing the dimension of the target distance space. In addition, the minimised MDS error (Methods) is small already at low target dimension. (c) Manhattan distance is highly correlated with Jaccard distance, yielding similar antigenic maps (not shown).

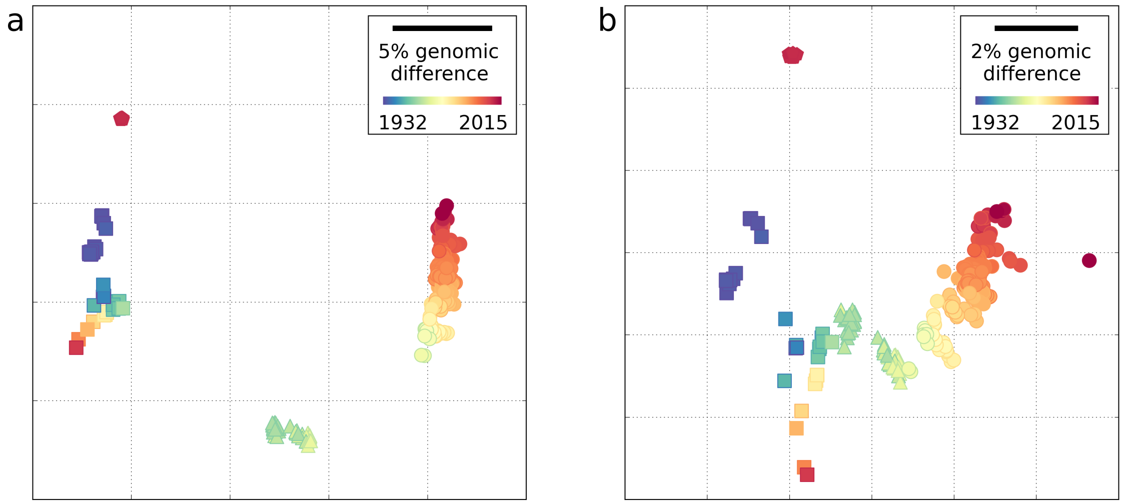

Supplementary Figure S4 MDS maps of IAV proteomes.

(a) The topology of proteome map differs considerably from the CTL antigenic map, with clear separation of subtypes (Fig. 1 and Methods). (b) Excluding the variable HA and NA proteins from the analysis brings the subtypes closer together, but the map remains different from the map based on epitopes. $R^2 = 0.99$ in (a) and $R^2 = 0.97$ in (b).

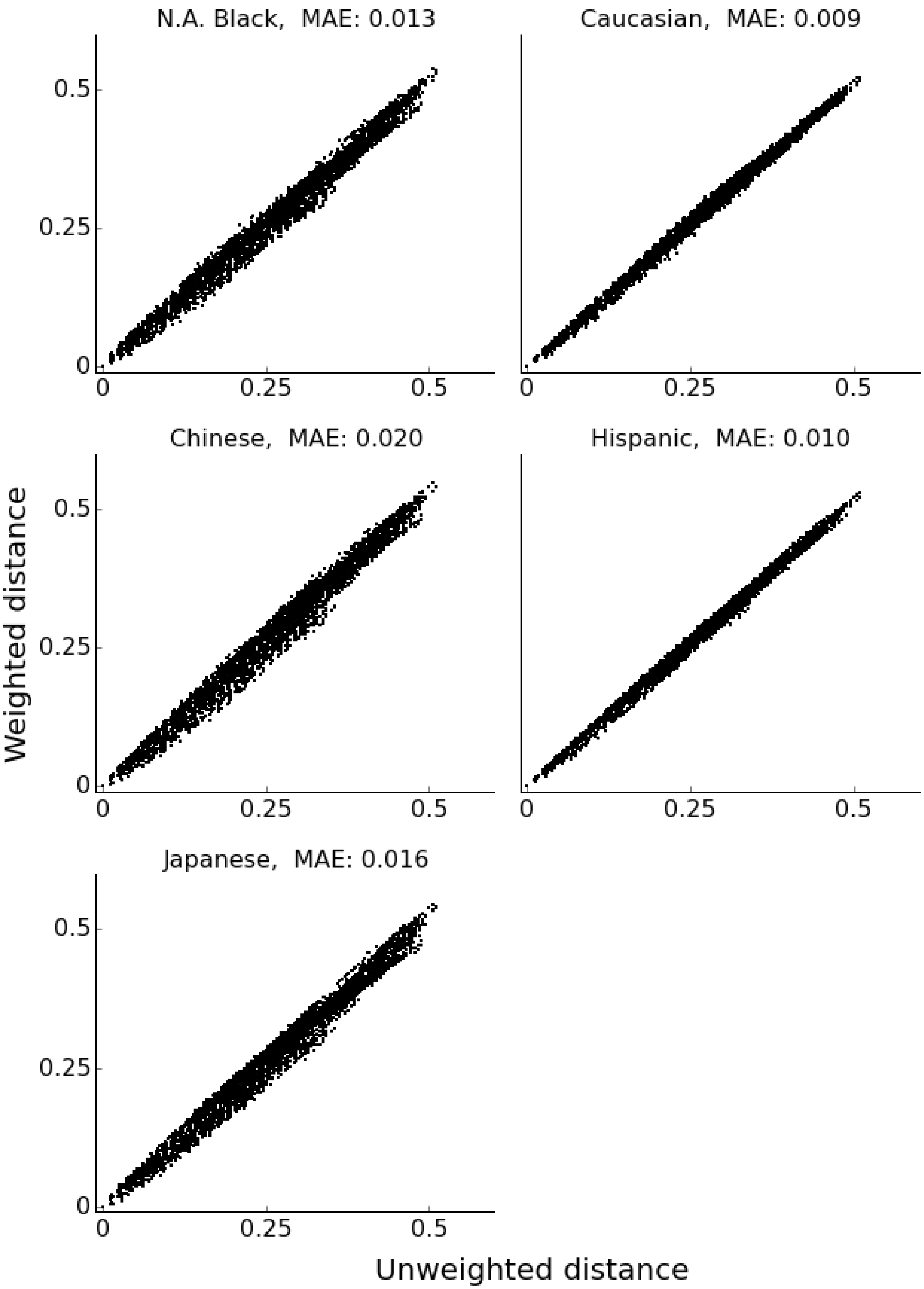

Supplementary Figure S5 Antigenic distances weighted with HLA frequencies of main ethnicities.

See next page for caption.

Supplementary Figure S5 Antigenic distances weighted with HLA frequencies of main ethnicities.

To test the robustness of the antigenic distance analysis to population immunodominance, we weighted the contribution of each epitope by the corresponding HLA supertype frequency in five main ethnicities (Black, Caucasian, Chinese, Hispanic, and Japanese)[1]. HLA supertype weighting has a minor effect on the pairwise virus distances for all ethnicities. This indicates that the epitope data set covers worldwide frequently occurring HLAs. MAE: mean absolute error compared to the diagonal.

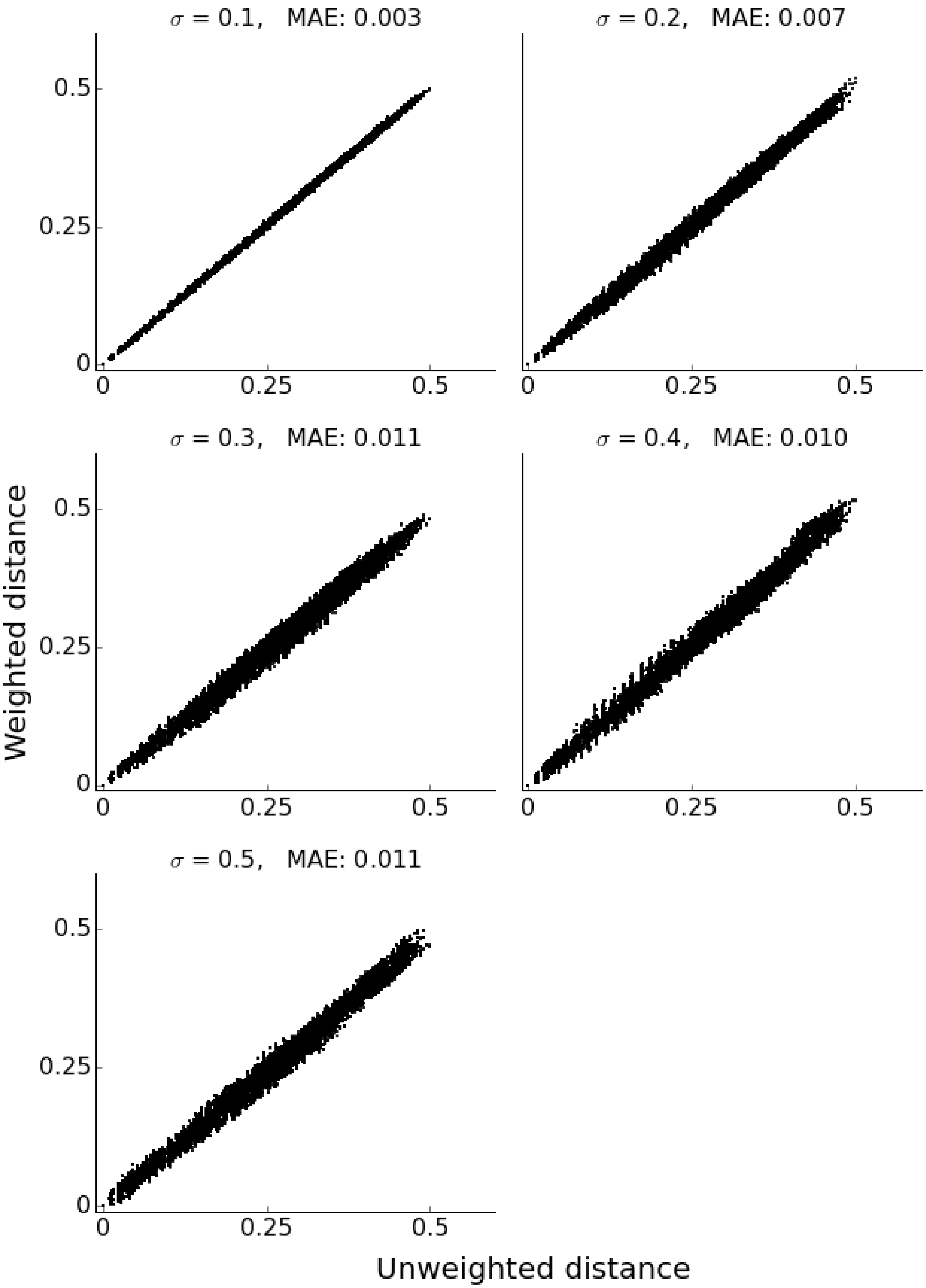

Supplementary Figure S6 Robustness of viral distances to immunodominance.

See next page for caption.

Supplementary Figure S6 Robustness of viral distances to immunodominance.

The relative immunodominance of individual epitopes is unknown. To test how variability in the relative immunodominance influences the antigenic map, we used a random weighting for each epitope by sampling from a (truncated) normal distribution with unit mean and five different standard deviations (σ), and compared weighted and unweighted distances. Even for large variability (σ = 0.5) we obtain only a small difference between weighted and unweighted distances (mean absolute error (MAE) = 0.011). This indicates that without knowing the exact relative immunodominance of the epitopes, the overall evolutionary pattern is accurately obtained using unweighted distances.

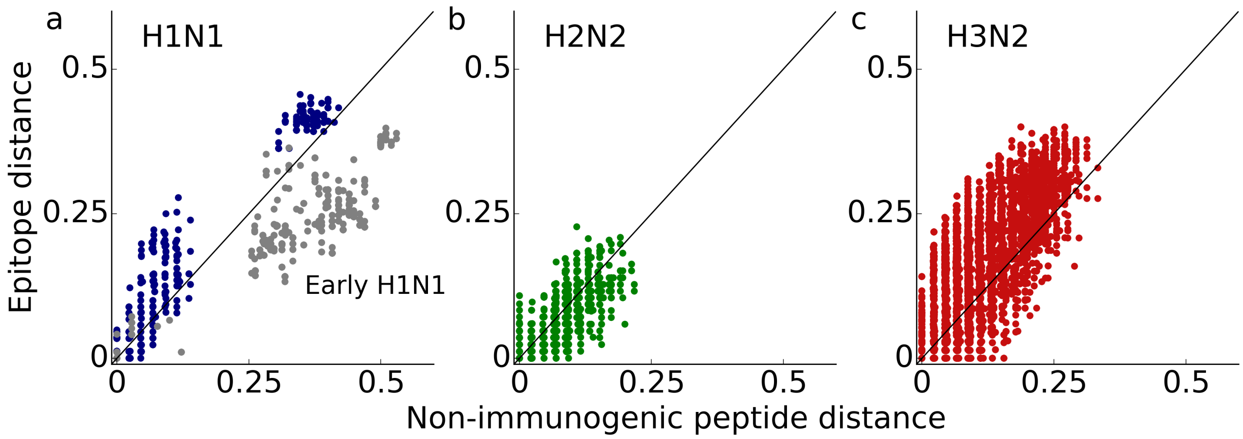

Supplementary Figure S7 Evolution of CTL epitopes versus non-immunogenic peptides. The distances between all viruses based on CTL epitopes are larger than the distances based upon non-immunogenic HLA-binding peptides. We obtained the (normalised) pairwise virus distances of H1N1 (a), H2N2 (b), and H3N2 (c) viruses using 134 CTL epitopes and 62 non-immunogenic peptides (Methods). A small number of H1N1 viruses from the years 1932-1935 differ more in their non-immunogenic peptides than in their CTL epitopes (grey dots). The H2N2 subtype circulated too shortly (1957-1968) to find a significant difference between epitope and non-immunogenic peptide distances. See Supplementary Fig. S8 for a statistical analysis of the comparison in H3N2.

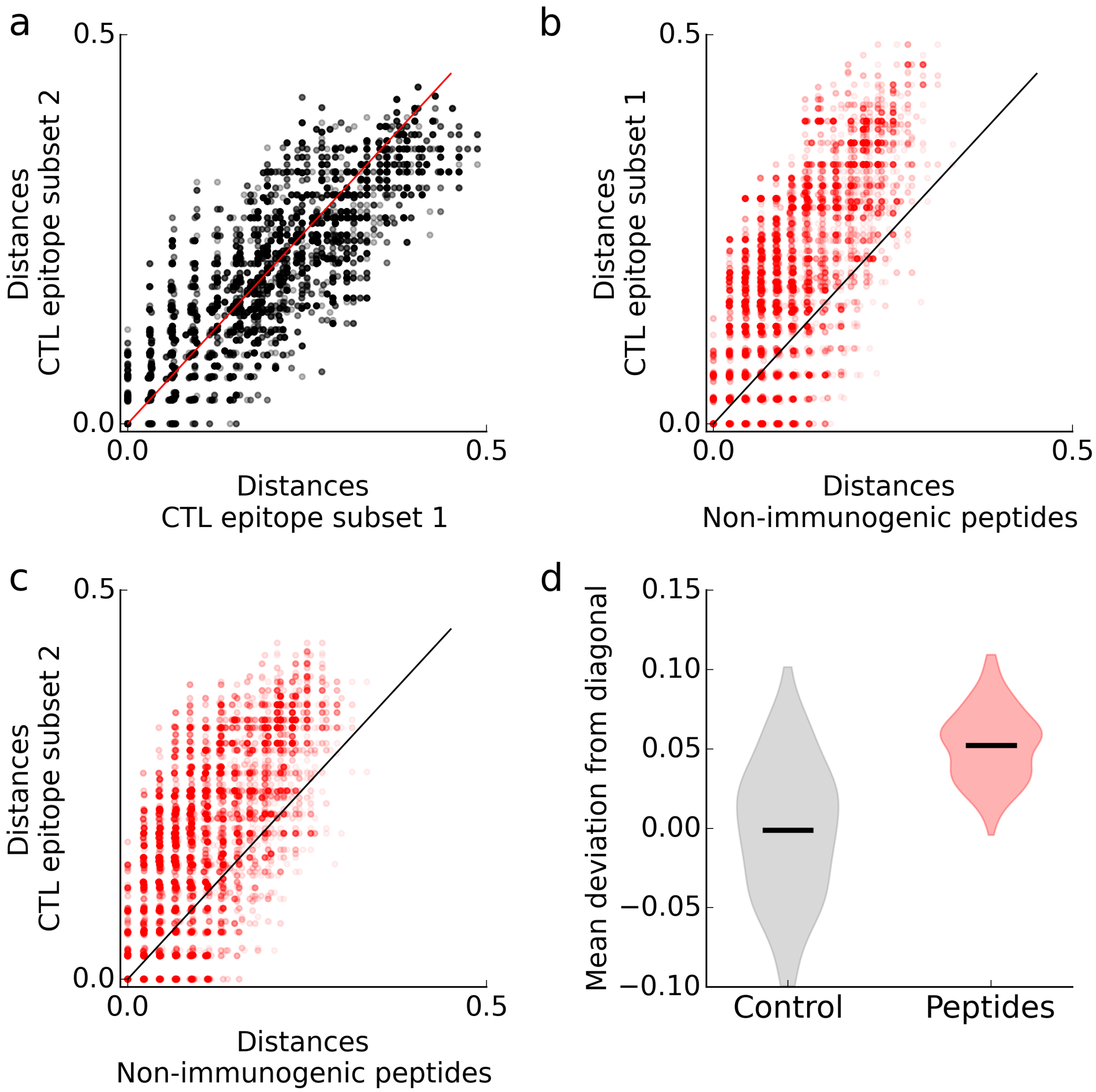

Supplementary Figure S8 Statistical analysis of the evolution of CTL epitopes versus non-immunogenic peptides in H3N2.

See next page for caption.

Supplementary Figure S8 Statistical analysis of the evolution of CTL epitopes versus non-immunogenic peptides in H3N2.

To show that CTL epitopes evolve faster than non-immunogenic peptides, i.e. distances between viruses based on CTL epitopes are on average larger than those based on non-immunogenic peptides, we randomly divide the set of CTL epitopes into two equally sized subsets with as many epitopes as the number of non-immunogenic peptides (i.e. 62). We compare the CTL epitope subsets with each other (a), and with the non-immunogenic peptides (b-c), and find that distances based on the CTL epitope subsets are larger than for the non-immunogenic peptides. (d) Repeating this analysis 100 times, we find a significant difference between comparisons among the subsets (control) and the comparisons with the subsets and the set of non-immunogenic peptides (peptides), measured as the average difference between each data point and the diagonal ($P < 0.00001$, Mann-Whitney U test). Notice that not every epitope is used in each sample, as the number of CTL epitopes (134) is more than twice the size of non-immunogenic peptide set (62).

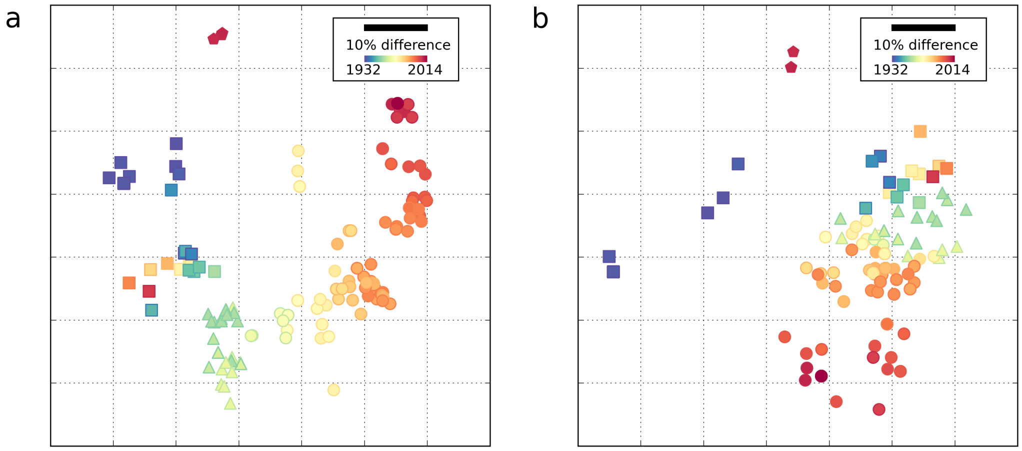

Supplementary Figure S9 MDS map of non-immunogenic peptides.

(a) Antigenic map as in Fig. 1 constructed using the confirmed CTL epitope compendium using unscaled distances (Methods). (b) The map constructed using pairwise virus distances obtained using 62 non-immunogenic peptides contrasts with the antigenic map in (a). In the map of (b), most viruses are close, with the exception of early H1N1 and recent pH1N1 viruses. Only the early H1N1 viruses (isolated before 1935) and recent pH1N1 differ substantially due to reassortments. The non-immunogenic peptides do not elicit an immune response in any assay performed (Methods). The two-dimensional antigenic map in (b) explains most of the variance ($R^2 = 0.96$).

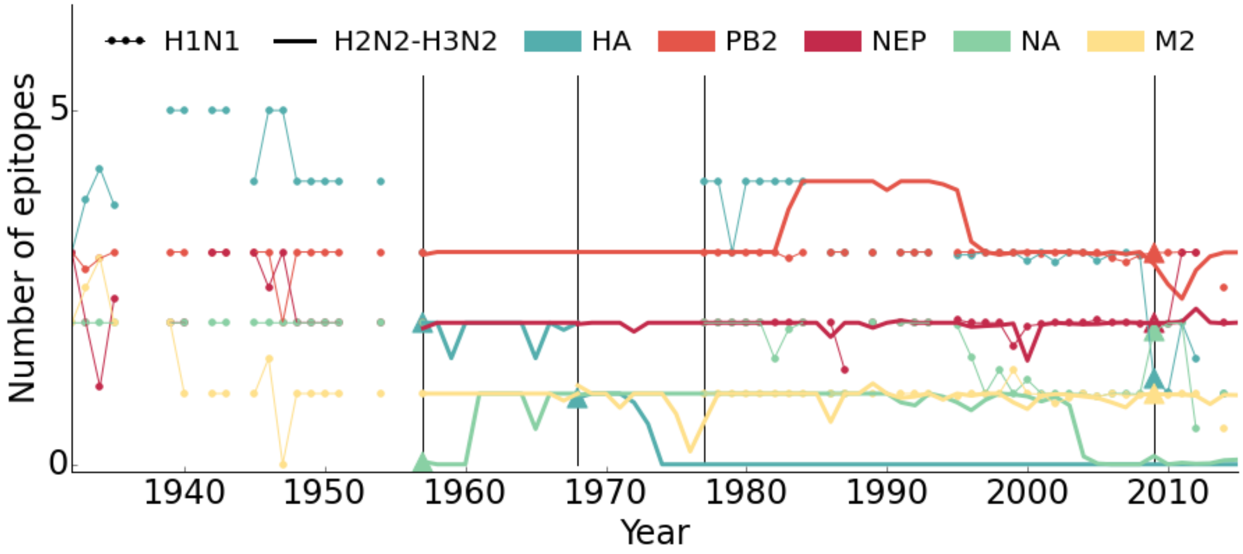

Supplementary Figure S10 CTL epitopes in IAV proteins containing small number of epitopes.

Time series of the number of CTL epitopes in proteins that harbour at most five epitopes. The number of epitopes in HA and NA decreases over time. Triangles mark reassortment events. See Fig. 2b for the number of CTL epitopes over time in the other proteins.

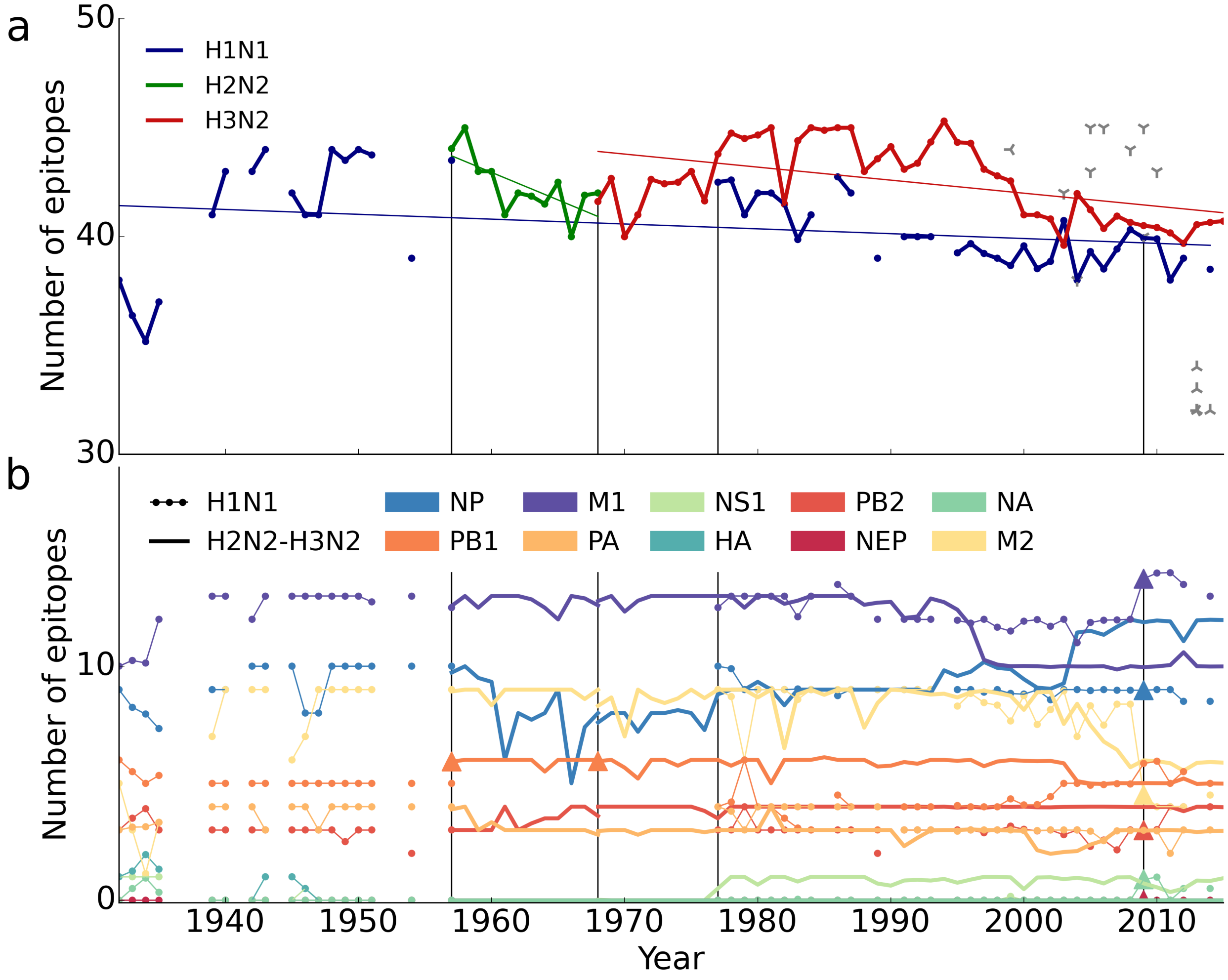

Supplementary Figure S11 Dynamics of non-immunogenic peptides.

(a) No significant adaptive decrease is observed in the number of peptides per virus that are not immunogenic in H1N1, H2N2, and H3N2 ($P = 0.45$, 0.32, and 0.49, respectively; Methods). The decrease is 0.022 ± 0.011, 0.25 ± 0.08, and 0.06 ± 0.02 peptides per year ($\pm$SEM) for H1N1, H2N2, and H3N2, respectively. (b) The number of non-immunogenic peptides per virus for each protein is constant over time, with only small changes in NP, M1, and M2 in the past two decades. We use peptides of IAV origin that are empirically tested and have a negative CTL response in all assays performed, and compute the occurrence in the complete virus set (Methods).

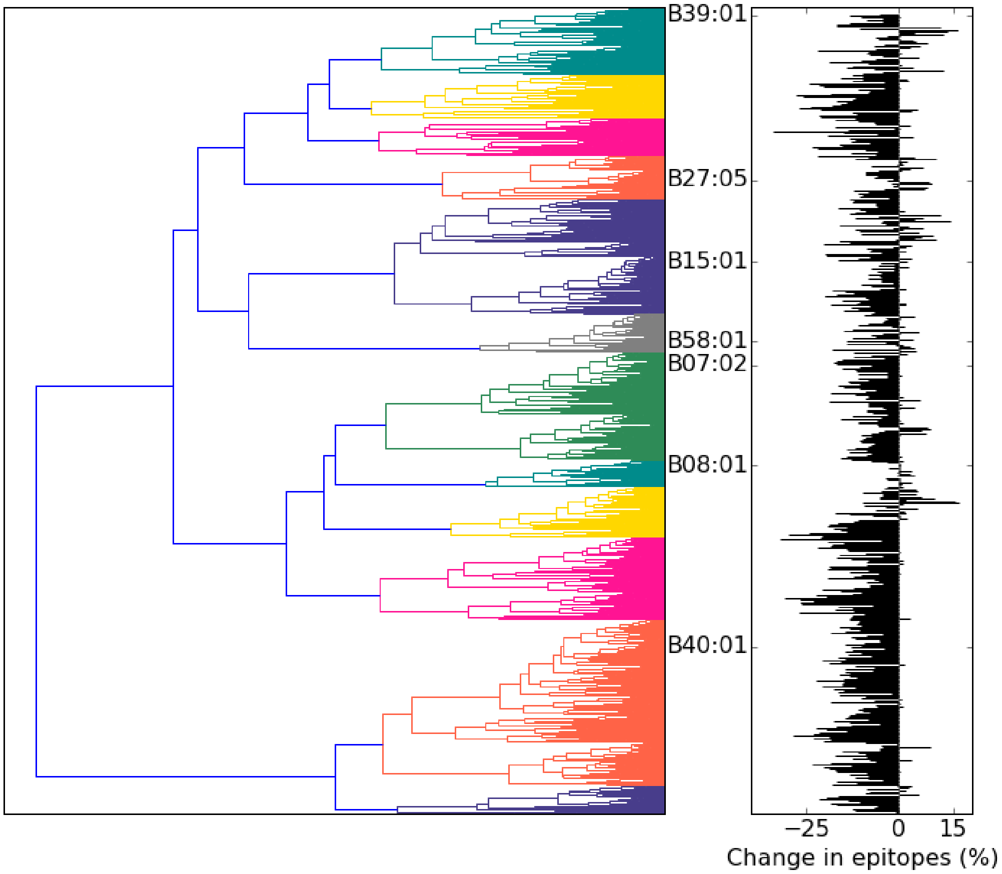

Supplementary Figure S12   Prediction-based cluster analysis of HLA-B alleles. We find a decrease in the number of predicted epitopes for the full HLA spectrum, indicating that a decreasing trend exists for HLA alleles with distinct binding motifs. The dendrogram (left) is based on Jaccard distances between HLA-B alleles using the 5% top binders in NP of H3N2 viruses. For each HLA allele the change in the number of predicted epitopes (top 5%) in H3N2 in the period 1968-2015 is indicated on the right. We labeled the 7 HLA-B supertype representatives, and set the cluster threshold such that no two supertype representative are in the same cluster. The dendrogram is constructed using the UPGMA algorithm.

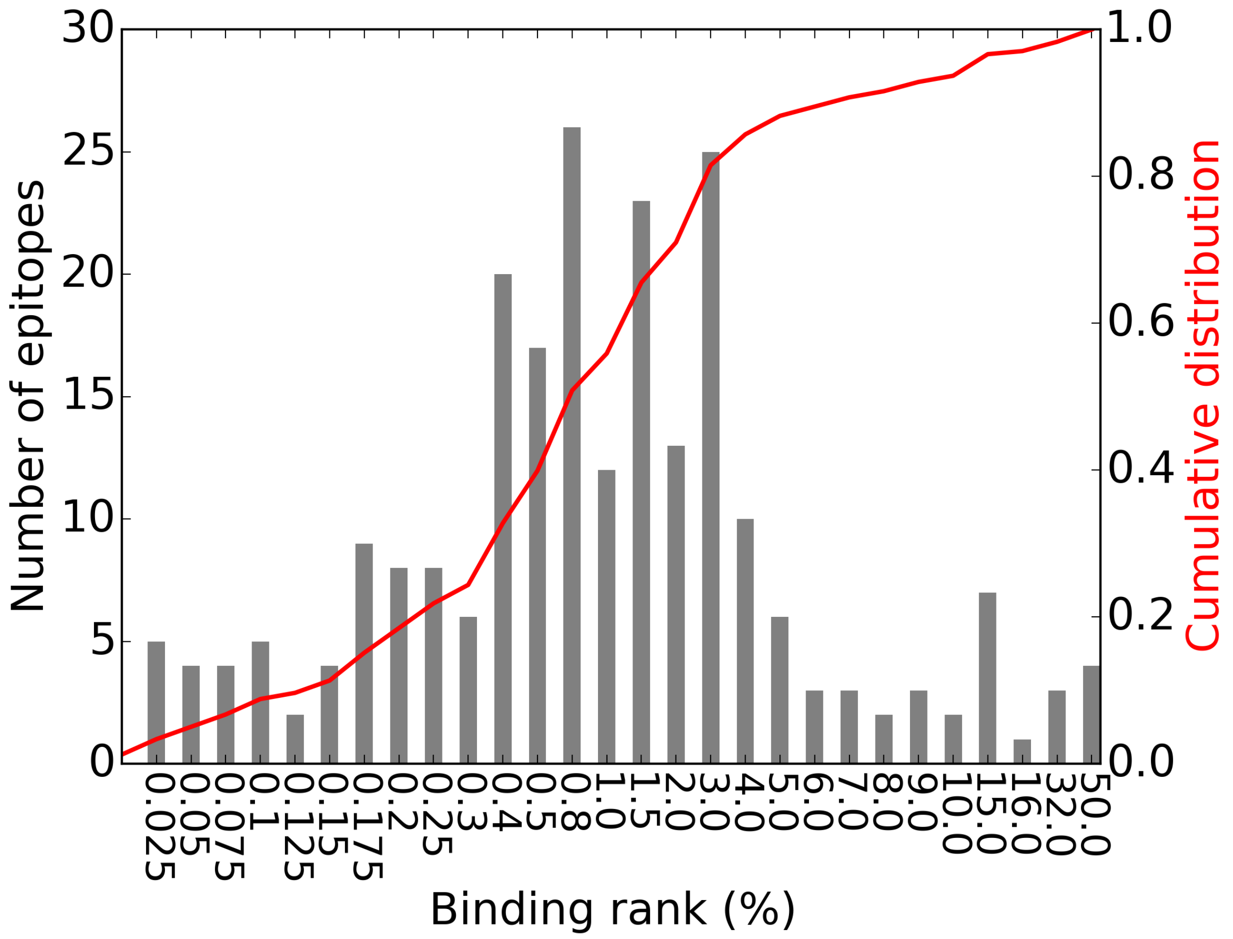

Supplementary Figure S13 Predicted HLA binding affinity of the empirical epitopes. We use the prediction tool netMHCpan 2.8[2] to predict the binding affinities of the epitopes obtained from IEDB, and plot the number of epitopes in various binding rank bins. The binding of all HLA-epitope combinations are considered, when multiple HLA alleles per epitope are reported in IEDB.

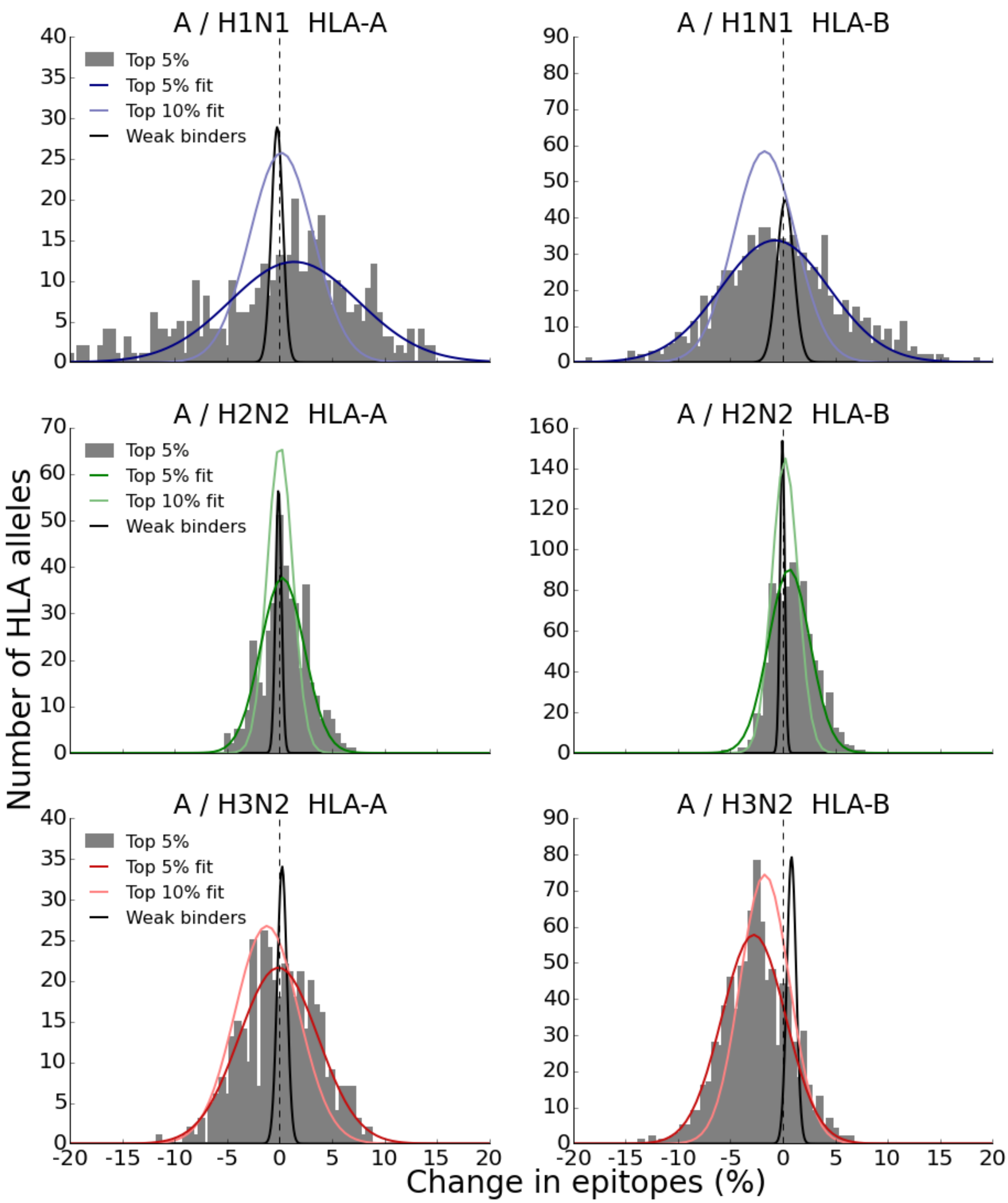

Supplementary Figure S14 Change in predicted top binders in H1N1, H2N2, and H3N2.

See next page for caption.

Supplementary Figure S14 Change in predicted top binders in H1N1, H2N2, and H3N2. Aggregating the proteins NP, M1, and PB1 that cover most empirical epitopes (Fig. 2b), we find a small decrease in the number of strongly binding peptides for most HLA-B alleles in the subtypes H3N2 (P = 0.069; mean change $\hat{\mu}$ = -3.6% (95% CI:-7.5%,0.3%) over the study period). We find no decreasing trend in H1N1 and H2N2, probably because the latter subtype only circulated for a decade and because H1N1 evolves slower (Fig. 2). The top 5% best binding peptides (relative to the default netMHCpan reference data set) is shown in a bar plot. Fitted Gaussian curves belong to the thresholds $\leq$5%, $\leq$10% (top binders), and $\geq$50% (weak binders). We performed the predictions using all viruses of H1N1, H2N2, and H3N2 in the periods 1977-2008, 1957-1968, and 1968-2015.

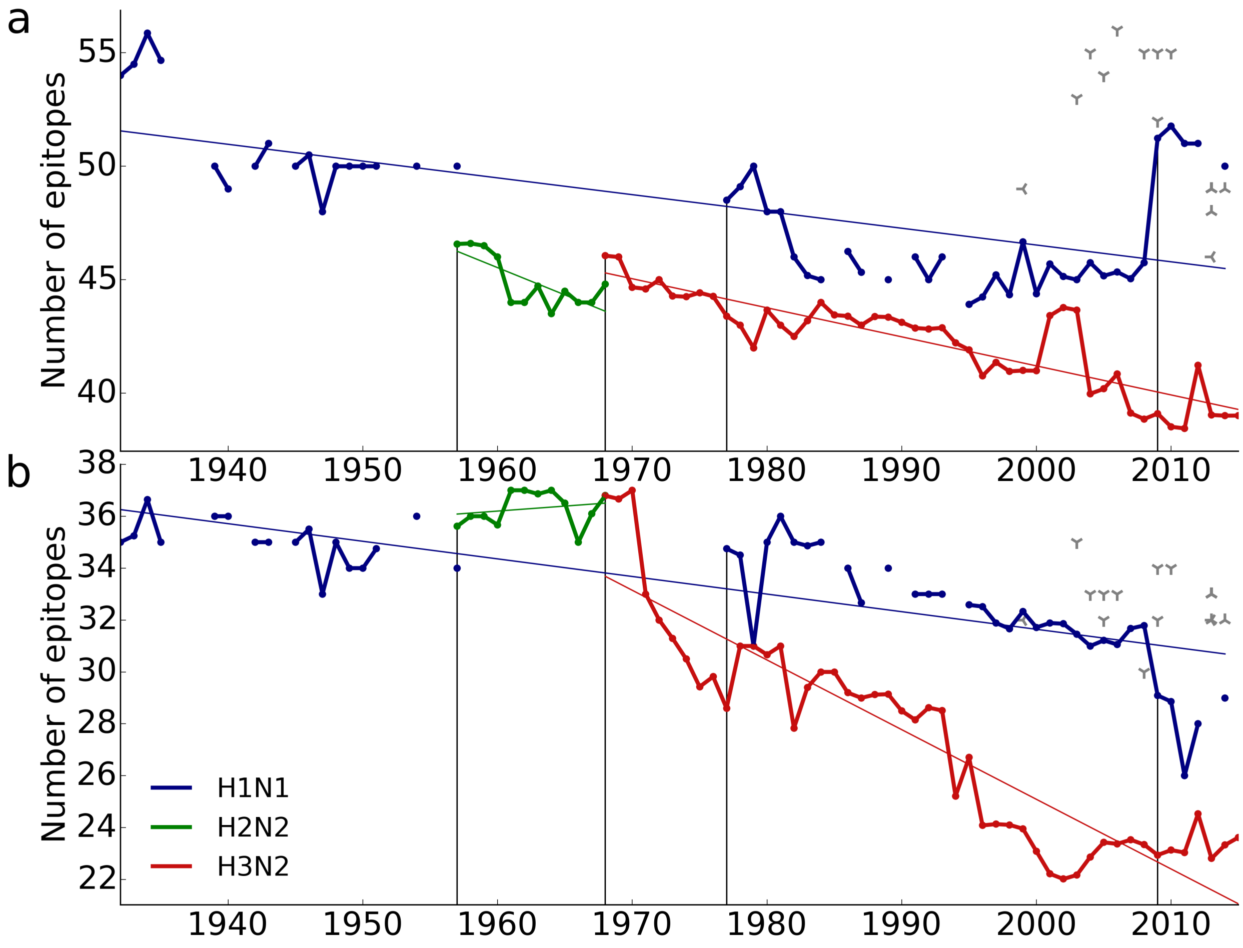

Supplementary Figure S15   Dynamics of CTL epitopes per HLA locus.

The number of CTL epitopes per virus in H1N1, H2N2, and H3N2 for HLA-A (a) and HLA-B (b) alleles. In H1N1, there is a decreasing (non-significant) trend for HLA-A and HLA-B. The year-to-year decrease is most pronounced in H3N2 (P = 0.045 and P = 0.093 for HLA-A and HLA-B, respectively; Wilcoxon signed-rank test; Methods). Avian viruses are of H5N1 (⅄), H7N9 (Y), and H9N2 (≺) subtypes.

Table S1 Characteristic influenza A virus set.

Table S2 Cytotoxic T lymphocyte epitopes in human influenza A viruses.

Table S3 Non-immunogenic peptides in human influenza A viruses.

Table S4 Human CTL epitopes in avian IAVs.

1. Sette, A. & Sidney, J. Nine major HLA class I supertypes account for the vast preponderance of HLA-A and -B polymorphism. *Immunogenetics* **50**, 201–212 (1999).

2. Hoof, I. *et al.* NetMHCpan, a method for MHC class I binding prediction beyond humans. *Immunogenetics* **61**, 1–13 (2009).
